# Strabismus

**Published:** 1890-10

**Authors:** A. Bethune Patterson

**Affiliations:** Late Assistant to the Royal London Opthalmic and Golden Square Throat Hospital, London, England


### STRABISMUS.

BY A. BETHUNE PATTERSON, M. D.,

Late Assistant to the Royal London Opthalmic and Golden Square Throat Hospital, London, England.

Strabismus, "squint" or "cross" eye, as it is commonly called in common parlance, is a want of equilibrium in the eye muscles—one eye being fixed upon an object, the other deviates, the axis of vision directed away from the point of fixation. This condition of the eyes is to-day absorbing the attention of the most learned eye surgeons.

I shall not attempt to discuss the various theories that are advanced as to the cause of strabismus. It is beyond the scope of this brief article to enter upon this very intricate and interesting subject. My only excuse in bringing this matter before the general practitioner is to impress upon them the importance of early recognition and treatment of strabismus that shows itself in young hyperopiæ, generally at an age when they begin to fix their eyes upon small and near objects. First it is periodic, and alternating after a while—the time varies when the strabismus becomes fixed. The child soon learns to suppress the image in the deviating eye, the deplopid or double vision, which, up this time, must cause considerable annoyance, now disappears, leaving the child virtually with one eye—binocular vision is at an end. This faculty, which is only enjoyed by man, once lost will tax the ingenuity of the most skillful physician to restore.

In high degrees of hypermatropea there will be found an excessive amount of convergence, on observing near objects, and it is known that for every meter-angle of convergence there is one dioptri of accommodation. The physiological relation existing between these functions is lost in hypermatropea at the expense of normal or binocular vision. One eye rotates inward, while the other fixes, and strabismus is established. On the restoration of the normal physiological condition, viz.: the correlation of accommodation and convergence depends our success in relieving the deviation, and as habit and exercise play an important part in the production of the normal movements and position of the eyes, no time should be lost in commencing treatment. These children are generally first seen by the family physician, who too often falls into the error of advising parents to wait, that it may be the child will eventually outgrow the squint, or what is equally hazardous, they advise a postponement of surgical interference until the child is eight or ten years old. I cannot be too emphatic in my disapproval of this advice. My experience in the hospitals of New York and London, and in private practice has convinced me that a large number of these children, if taken in time and treated as I shall hereafter suggest, can be cured without surgical interference. I regret to say that too often these children fall into the hands of surgeons who appear to be unmindful of the above facts, and resort to tenotomy. Apparently these operations are successful, the parents' anxieties are relieved, at least for a time. Are these eyes straight? Have the eyes been restored to their normal function? I say no. The eye is virtually blind, binocular vision does not exist, much yet remains to be done before the patient is dismissed. Only a part of the way has been gone in the treatment or cure. In the course of time a large percentage of these eyes turn out, the last stage is worse than the first. The promiscuous cutting of the internal recti muscle is to be deprecated. I do not wish to be understood as being opposed to surgical procedures in strabismus; not at all; but I do strenuously oppose the unscientific cutting the internal recti muscle. Time enough to resort to tenotomy when methods of a conservative character have been exhausted. During my stay with Dr. H. Knoff, of New York city, I observed that it was his custom to resort to the non-surgical treatment in nearly all young children, and, I will state, with encouraging results.

The treatment to be pursued is, first, to ascertain the degree of hypermatropea, with the accommodation set at rest by the instillation of atropine. We now proceed with glasses and test-type at a distance, or with retenoscopy. Then, by a course of atropine, ranging from a few months to a year or more, and a full correction of ametropia, we hope to be rewarded by seeing the eyes take up their natural position and function. In prescribing glasses in young children, it will tax the skill and patience to refract them correctly. Many of them have not yet learned their letters, while others give very unsatisfactory answers. I prefer retenoscopy for determining the correcting glass. Dr. Stephens says: "The correction of squint with the view of obtaining perfect binocular vision throughout the whole range of vision, instead of being one of the easiest of surgical operations, is a procedure demanding the supreme ability of the accomplished observer and the highest skill of the dexterous operator." It is especially so in those cases where the internal recti has been divided. I have found these cases the most tedious, frequently necessitating further surgical interference. The orthoptic training consists of daily exercising the muscles of the eye, endeavoring to stimulate the desire for binocular vision. This is accomplished by the assistance of a candle and prisms, and what is also indispensable, an ordinary stereoscope, and in the place of pictures or views, geometrical figures are used. Landolt employs two vertical lines—one above and the other below the same horizontal line. A candle placed at a distance of eighteen or twenty feet, and the patient made to note the position of the two flames. With many of these cases they declare they only see one flame, and only after repeated trials they are brought to recognize the double images. Now, with prisms we endeavor to assist the patient in bringing the flames together. This training must be persevered in daily for months, until fusion of images can be accomplished without the aid of prisms. When this has been accomplished we can say that the strabismus has been cured, and not till then.

---

PERCHLORIDE OF IRON IN LEUCORRHŒA.—Of all remedies for simple leucorrhœa, the old tincture of perchloride of iron is the best, combined with hyoscyamus, opium, hop or Indian hemp, when the mucous membrane is in a state of irritation. Tepid or cold water injections, cold hip baths, etc., are useful local applications, with rest, and avoidance of occupations involving prolonged standing or pedal exercise.

Sometimes tannin, zinc or alum are valuable additions to the injections. When the discharge emanates from the glands of the os uteri, local applications of belladonna and bicarbonate of potash are serviceable, two ounces of tincture and a teaspoonful of the alkali to a pint of water.—Editorial in *Pharm. Era.*

---

A NEW METHOD FOR AROUSING PATIENTS FROM CHLOROFORM NARCOSIS.—We learn authoritatively that a homœpath of this city uses no other method for restoring patients to consciousness from chloroform narcosis than the dilatation of the sphincter âni muscles, which is accomplished by thrusting three fingers up the rectum and dilating it in a most vigorous manner. We understand that in the numerous instances in which this novel method has been tried the patients invariably gave a Comanche "whoop-pee-a-whoop-pa-o-oo-ugh" yell that startled the natives for several blocks. It is claimed this discovery was made by observing the ease with which patients recover from chloroform in operations on the rectum.—*Texas Health Journal.*

